# The role of short-time work and discretionary policy measures in mitigating the effects of the COVID-19 crisis in Germany

**DOI:** 10.1007/s10797-022-09738-w

**Published:** 2022-05-23

**Authors:** Michael Christl, Silvia De Poli, Tine Hufkens, Andreas Peichl, Mattia Ricci

**Affiliations:** 1 Fisacl Policy Analysis Unit (B2), European Commission (DG JRC), Seville, Spain; 2 ifo Institute and LMU, Munich, Germany

**Keywords:** COVID-19, EUROMOD, Microsimulation, STW, Automatic stabilisers, D31, E24, H24

## Abstract

In this paper, we investigate the impact of the COVID-19 pandemic on German household income in 2020 using a micro-level approach. We combine a microsimulation model with novel labour market transition techniques to simulate the COVID-19 shock on the German labour market. We find the consequences of the labour market shock to be highly regressive with a strong impact on the poorest households. However, this effect is nearly entirely offset by automatic stabilisers and discretionary policy measures. We explore the cushioning effect of these policies in detail, showing that short-time working schemes and especially the one-off payments for children are effective in cushioning the income loss of the poor.

## Introduction

The COVID-19 crisis led to a strong fall in economic activity and, consequently, to a severe drop in GDP and an increase in unemployment across Europe. In Germany, the recession in 2020 was about as significant as the financial crisis in 2008/2009, with a drop in GDP of about 5%. But also the labour market impact of the COVID-19 crisis was severe. While in February 2020 only about 133,000 workers were registered in short-time work (STW), this number rose sharply in March and April, with almost 6 million workers being moved to this scheme.^1^ To counteract the consequences of the COVID-19 pandemic, the German government strengthened and extended the existing STW scheme that already proved its worth during the financial crisis of 2008/2009. Moreover, several discretionary policy measures (DPM) were introduced. Both, STW and DPM had the goal to cushion against the income loss of households and to prevent a strong drop in private consumption. While the macroeconomic impact of the COVID-19 crisis has already been well documented (almost in real-time), evidence of the distributional impact on household income at the micro-level is more limited. This is largely due to missing real-time micro-data. In this study, we address this issue by simulating the impact of COVID-19 on the labour market and on household income across the income distribution in Germany in 2020.

So far, the literature has employed two main approaches to overcome time lags in micro-data when assessing the impact of macroeconomic shocks. The first is a static approach that typically consists of updating the weights of individual observations in the micro-data to mirror up-to-date aggregate labour market statistics. For example, Almeida et al. (2021) use re-weighting techniques to update EU-SILC data and estimate the impact of COVID-19 on household income in EU countries. Beznoska et al. (2020) combine pre-crisis survey data with a questionnaire on subjective assessment of the labour market and the income loss of households via matching in order to estimate the impact of COVID-19 on household income in Germany. The second is a dynamic approach that employs labour market transition techniques to change the employment status of individuals in the micro-data to replicate labour market developments. For individuals who change their labour market status, taxes and benefits are updated consistently with the tax-benefit rules in place in each country. The key advantage of the labour market transition approach, that we also use in our analysis, with respect to re-weighting/matching is that this approach does not need to assume that the characteristics of the affected subgroup of the population (e.g. individuals in STW) remain unchanged. Moreover, by re-calculating taxes and benefits, it can account for changes in legislation including, for example, benefit amounts or eligibility conditions. Such an exercise is conducted by Cantó et al. (2021), who use labour market transition techniques for a selection of EU countries. Christl et al. (2021a) also used the labour market transition approach to estimate the impact of COVID-19 related policy measures in a cross-country setup for all EU countries, showing that policy measures were cushioning substantially the income loss and the inequality increasing feature of the COVID-19 pandemic.^2^

In this paper, we use EUROMOD, the microsimulation model of the European Union, to analyse the impact of the COVID-19 crisis on employees in Germany, as well as the role of STW in cushioning its effects on household income. We extend the labour market transition approach using both administrative data on the use of the STW scheme from the Federal Employment Agency (“Bundesagentur für Arbeit”) as well as survey data from the HOPP database of the Institute of Employment Research (IAB) to estimate a probit model (based on a broad set of worker characteristics) to identify workers that move to STW schemes.^3^ These data cover the whole year 2020, and they are used to update the EU-SILC microdata underpinning EUROMOD. We then employ this model to investigate the impact of COVID-19 on household income across the income distribution in Germany and, thus, its effect on inequality and poverty. We find that the COVID-19 crisis had a large impact on household income across the whole spectrum of the income distribution, with the average drop of market income in excess of 3%. The effect is largely regressive, indicating a significant increase in income inequality. However, the regressive impact on income is nearly entirely offset by automatic stabilisers (such as unemployment benefits) and DPM introduced during the crisis (notably, child-related COVID-19 benefits and a tax allowance for single parents). We find that the STW scheme and DPM played a much greater role in stabilising the income of households located in the bottom half of the income distribution than of those sitting in the upper half. Indeed, in the absence of STW and DPM, richer households would have seen their disposable income mostly unaffected, while the poorest households would have faced the largest loss in disposable income.

Our work contributes to the literature along several lines. First, we simulate wage compensation schemes on a micro-level using a methodology of labour market transition based on micro-simulation techniques. This allows us to transit individuals to both unemployment and STW schemes. Second, we use detailed administrative data on the number of people in STW schemes and unemployment in 2020. For workers in STW schemes, these data also allow us to model the duration and reduction of working hours for a more precise assessment of the impact of the COVID-19 crisis on the German labour market. Third, we compare different modelling approaches for selecting individuals in the micro-data who change their labour market status in response to the crisis. We show that such a modelling choice substantially affects the estimation of the impact of the COVID-19 crisis across the income distribution. Contrary to the results found using traditional approaches (which are based on either re-weighting or stochastic labour market transitions), we find that using the extended labour market transition approach the impact of the COVID-19 crisis on disposable household income is quite similar in size across the income distribution. Fourth, we add to the discussion on the cushioning effect of STW and discretionary policy measures. In particular, we show that STW and DPM in Germany are increasing the income stabilising mechanism, especially for low-income earners.

The literature on the distributional impact of the Covid crisis for Germany is scarce. In addition to the few papers mentioned above, Bruckmeier et al. (2021) extend the approach of Bargain et al. (2012) and combine macro-and micro-modelling to nowcast the macroeconomic effect of COVID-19 in Germany in 2020. The key difference of their approach to our analysis is in the selection of individuals who experience a shock.^4^

Similarly to our analysis, they analyse the impact on the labour market and household income. They find a substantial decrease in gross labour income across the income distribution. However, the tax benefit system and discretionary policy responses to the crisis are able to cushion this effect, leading to a progressive effect of the COVID crisis. As we show in the Appendix, this would have also been the case in our analysis had we selected at random the individuals who experience the labour market transition in each sector of the economy. Instead, by using a probit model controlling for a broad set of worker characteristic, we can account for the fact that individuals in the lower part of the income distribution are more likely to enter in the STW schemes.^5^ This shows the importance of accounting for worker characteristics when simulating labour market transitions in micro data instead of using the sector of activity alone. Moreover, our analysis of income stabilisation effects as well as more detailed STW and DPM responses using counterfactual scenarios is novel compared to Bruckmeier et al. (2021).

The rest of the paper is structured as follows. Section 2 provides a brief description of the STW scheme and discretionary policy measures in Germany. Section 3 outlines our methodology as well as the data we employ for our analysis. Section 4 presents the results, while Sect. 5 offers some concluding remarks.

## Short-time work and discretionary policy measures in Germany

In this section, we briefly describe the features of the STW scheme and of the main DPM that were active during the COVID-19 crisis in 2020. The role played by these policies in cushioning the impact on household income will then be at the core of our analysis in the remainder of the paper.

### Short-time work scheme

The STW scheme has been an essential tool for containing the extent of job destruction in the ongoing COVID-19 crisis in Germany. This scheme has already been in place for many years; for example, it played an important role in cushioning the impact of the financial crisis of 2008–2009, see Bargain et al. (2012).

STW consists of a contributory benefit paid by the social security unemployment insurance. The benefit compensates employees for wage losses due to an involuntary decrease in working hours. All employees subject to social insurance contributions are entitled to the scheme if the employer applies (and qualifies) for reduced working hours. The benefit amount is calculated based on the difference in net earnings before and after the reduction of working hours. In more detail, the amount is set to 60% of the difference in net earnings for individuals without children and 67% for individuals with children. Importantly, the system of STW that existed before the pandemic was further expanded at the beginning of the pandemic, both in terms of access and of monthly rates. Specifically, employees who received the compensation for more than four months in 2020 (and whose working hours were reduced by at least 50%) had their compensation rate increased to 70% in the fifth and in the sixth month (77% for beneficiaries with children). From the seventh month, the rate increased to 80% for individuals without children and to 87% for parents. In addition, from March 2020, temporary employees were also entitled to the compensation. Employers could use the scheme already if 10% or more of their employees were affected by the lack of work, while before the pandemic this limit was set to 30%. Another change due to the COVID-19 pandemic is that the basic social security contributions to be paid by the employer are paid by the unemployment insurance. Finally, the German government decided to prolong the current scheme until December 2021, whereas originally this benefit was only paid for a maximum of 12 months.

In EUROMOD, the eligibility for the benefit is based on net earnings, the reduction of hours worked, the duration of the benefit and if the individual has children. The net earnings are calculated using a simplified tax schedule and simplified computations of social insurance contributions. The difference in earnings before and after the reduction of working hours is used to calculate the monthly benefit.^6^

Strictly speaking, the expansion of the STW enacted at the beginning of the pandemic should be considered a discretionary policy measure for its circumstantial and temporary nature. Therefore, a rigorous classification would require to separate the role of the structural component of the STW (i.e the part pre-existing the COVID crisis) from its extension (i.e the part introduced in response to the COVID crisis). However, in our study we aim at analysing the role played by the STW as opposed to the other policy measures introduced and the remaining parts of the tax-benefit system. We therefore consider all components of the STW policy as a whole and exclude them from the definition of the DPM below.

### Discretionary policy measures

On top of the extension of the STW scheme, several DPM were put in place in 2020 to alleviate the social consequences of COVID-19. One of their main goals was to protect the income of families with children. Indeed, this group was hit especially hard by the lockdowns, which imposed the closure of schools and the related need of homeschooling for children (as also highlighted by Blömer et al. 2021). In our analysis, we consider the most relevant (in monetary terms) of these policies,^7^ i.e: (i) the COVID-related child bonus, and (ii) the tax allowance for single parents.

The COVID-19 related child bonus is a one-off payment to support families with children. The same eligibility rules apply as for the standard child benefit in Germany. Consistently with the standard child benefit, the age limit is extended to 24 years for children who are still in tertiary education, and there is a limit on hours worked by the child.^8^ However, differently from the standard child benefit, the child bonus is not deducted from any means-tested benefits. The parents of the eligible child receive 300 euro per child. As discussed by Beznoska et al. (2020), this instrument is especially relevant for low-income families.

The tax allowance for single parents (“Alleinerziehendenentlastungsbetrag”), already existed before COVID-19 but was increased in 2020 and 2021. In more detail, the tax allowance was increased from 1,908 euro per year in 2019 to 4,008 euro per year in 2020 and 2021. The goal of this policy is to compensate single parents for the higher costs of living during the COVID-19 crisis.^9^

Finally, another important class of policies are those introduced to sustain self-employed and small businesses. These notably include the “Soforthilfe”, i.e. the immediate assistance program for small businesses and self-employed. “Soforthilfe” is a one-off benefit which was introduced to pay for current expenses and to compensate for the operating losses during the lockdown.^10^ Even though these measures are implemented in EUROMOD, we do not include them in our analysis because of the lack of information on operating losses and on the number of individuals using the scheme. This is clearly a drawback of our analysis, since, as highlighted by Graeber et al. (2021), self-employed are more likely to suffer an income loss due to COVID-19 than employees.

## Methodology and data

In this section, we discuss the methodology underpinning our analysis as well as the data we use to estimate our probit model and to calibrate the labour market transition in EUROMOD. In Sect. 3.1, we review the techniques the literature has employed to update micro-data. We explain why in times of rapid changes, such as the ongoing pandemic, our dynamic labour market transition approach is preferable to static approaches. We then provide a detailed description of the scenarios we simulate in our analysis and of the income stabilisation coefficient we assess in these scenarios. In Sect. 3.2, we then move to review the data we used for calibrating our scenarios and present the estimation of our probit model.

### Methodology

#### Extended microsimulation technique

The timely analysis of economic shocks is an important task for academics but is especially important for policy makers. At the time of writing, available micro-data for Germany do not include information on the effects of the COVID-19 pandemic in 2020. Given the substantial time lags in survey data, microsimulation models are largely used to assess the impact of rapid changes in the population and labour market conditions on income, poverty and inequality. This class of models, typically based on representative household data (or on administrative data), allow to assess the detailed impact of demographic changes and policy changes in a timely manner, as also highlighted by Immervoll et al. (2006) and Peichl (2009).

Generally speaking, two main approaches are employed to introduce demographic and labour market shocks in the micro-data. The first approach is a static technique, which typically consists of updating individual observations in the micro-data to mirror up-to-date labour market statistics. This is typically achieved by re-weighting, that is, by updating the weights of individual observations in the micro-data to meet target statistics, such as the updated unemployment rate (see e.g. Creedy, 2004; Dolls et al., 2019; Almeida et al., 2021). Alternatively, this can be achieved by matching procedures that combine older micro-data with individual-level information on the effects of a labour market shock, for example, income-loss questionnaires as in Beznoska et al. (2020). However, such a static approach has significant shortcomings when the characteristics of the relevant population groups (e.g. the unemployed population) change substantially. That is because by re-weighting/matching existing observations, it implicitly assumes that the characteristics of this subgroup of the population remain largely unchanged. Moreover, policy changes, such as a change in the benefit amount or the eligibility conditions, cannot be taken into account in detail. The second approach is a dynamic technique, which is based on the implementation of the labour market transition of individuals, as explained in detail in Gasior and Rastrigina (2017). More specifically, this method alters the employment status of individual observations in the micro-data consistently with up-to-date statistics and updates their incomes and labour market variables accordingly. For individuals affected by the transition, taxes and benefits are recalculated consistently with the tax-benefit rules in place in a given country. For example, if an individual “is transited” from employment to unemployment, the underlying microsimulation model will apply the policy rules in place to determine her unemployment benefits entitlement, as well as to reevaluate her entitlement for any other benefits and liability for taxes. This dynamic approach is preferable to the static one especially in times of rapid changes in the labour market and when policy changes have taken place. Hence, it is arguably better suited for the analysis of the COVID-19 crisis.

For our empirical analysis, we use the data from the EU Statistics on Income and Living Conditions (EU-SILC) in combination with EUROMOD for the simulation of taxes and benefits. EUROMOD is the microsimulation model of the European Union (see Sutherland and Figari, 2013 for more information), and its latest version features the policy rules in force in 2020 and input data based on EU-SILC 2018. Monetary values in input data, for example incomes, have been uprated to the 2020 policy year using appropriate uprating factors.^11^ We then simulate the COVID-19 labour market using the Labour Market Adjustment (“LMA”) add-on, which implements in EUROMOD the labour market transition techniques described above. The detailed description of the Add-on can be found in the technical annex of Christl et al. (2021a).^12^ In more detail, similar to what was done by Christl et al. (2021a) in a cross-country framework, we update the labour market by adjusting the labour market characteristics and market incomes of the selected individuals. Using EUROMOD, we are then able to simulate taxes and benefits taking into account the changes in the labour market.

In order to select the observations which experience a labour market shock, we estimate a probit model that identifies the likelihood that individuals change their labour market status following the COVID-19 shock. Given the nature of the COVID-19 shock, the two relevant transitions are: (i) from employment to unemployment, and (ii) from employment to STW. In the first case, employment income is adjusted proportionally to the numbers of months left in employment. The add-on then generates the variables needed to assess the entitlement to unemployment benefits, such as the contributions paid towards the unemployment insurance in the two previous years. In the second case, employment income and the number of months in employment are adjusted proportionally in consideration of the time spent in STW.^13^ Due to lack of information on self-employed, our analysis is limited to employees.

#### Simulation scenarios in detail

Using EUROMOD, we simulate the impact of COVID-19 on disposable household income under three different scenarios. Let *t* be the tax-benefit function that depends on: (i) the tax-benefit structure, *P*, which may include the COVID-related policy ($P^{Covid}$), or may not ($P^{NoCovid}$), and (ii) the labour market condition, *LM*, including COVID-related labour market transitions ($LM^{Trans}$), or not ($LM^{NoTrans}$). We can then define our three scenarios as follows:

- *Baseline (no-COVID-19 scenario)* Our baseline is a hypothetical COVID-19 free scenario. It is based on the 2020 tax-benefit system in EUROMOD that excludes DPM, as well as any transition to STW. For this scenario, we use EUROMOD underlying input data without introducing any changes in the labour market situation. In more formal terms, $t(P^{NoCovid}_{2020},LM^{NoTrans}_{2020})n$.
- *COVID-19 scenario* The COVID-19 scenario is based on the 2020 tax-benefit system, including the STW scheme and the DPM introduced in response to the pandemic. In this scenario, we update the micro-data using labour market transition to account for the labour market shock generated by the COVID-19 crisis. In more formal terms, $t(P^{Covid}_{2020},LM^{Trans}_{2020})$.
- *COVID-19 scenario w/o STW and DPM* This counterfactual scenario simulates the COVID-19 shock assuming that STW scheme and DPM were not in place in 2020. In this scenario, we therefore assume the same reduction in working hours as in the “COVID-19 scenario” above, but with workers transiting to unemployment instead of entering STW. More specifically, a corresponding number of workers in STW, in full-time equivalent terms, are assumed to move to unemployment instead. In more formal terms, $t(P^{NoCovid}_{2020},LM^{Trans}_{2020})$.

To measure the direct impact of COVID-19, while accounting for the cushioning effect of the STW scheme and of the DPM, we analyse the changes between the first two scenarios, $\Delta ^{PM}$, defined as:

1 $$\begin{aligned} \Delta ^{PM}_X=X\Bigl (t\bigl (P^{Covid}_{2020},LM^{Trans}_{2020}\bigr )\Bigr )-X \Bigl (t\bigl (P^{NoCovid}_{2020},LM^{NoTrans}_{2020}\bigr )\Bigr ). \end{aligned}$$

Where the function, *X*, can either return a certain income concept (disposable income or market income) or an inequality/poverty indicator (such as the at-risk-of-poverty rate or the Gini coefficient).

To analyse the impact of COVID-19 in absence of STW and DPM, $\Delta ^{NoPM}$, we compare instead the first and the third scenario:

2 $$\begin{aligned} \Delta ^{NoPM}_X=X\Bigl (t\bigl (P^{NoCovid}_{2020},LM^{Trans}_{2020}\bigr )\Bigr ) -X\Bigl (t\bigl (P^{NoCovid}_{2020},LM^{NoTrans}_{2020}\bigr )\Bigr ). \end{aligned}$$

Finally, to evaluate the cushioning effect of the STW scheme and DPM, we compare the impact of COVID-19 with and without these policies in place, respectively, $\Delta ^{PM}_X$ and $\Delta ^{NoPM}_X$.

#### Income stabilisation coefficient

To assess the income stabilising effect of the German tax-benefit system, as well as of any of its components, we follow the approach of Dolls et al. (2012), that was also employed by Christl et al. (2021a) in a cross-country set up and by Kyyrä et al. (2021) for Finland. They define the Automatic Stabilising Coefficient (ASC) as:

3 $$\begin{aligned} ASC = 1 - \frac{\sum _i \Delta Y^{D}_{i}}{\sum _i \Delta Y^{M}_{i}} = \frac{\sum _i \Delta Y^{M}_{i} - \sum _i \Delta Y^{D}_{i}}{\sum _i \Delta Y^{M}_{i}}, \end{aligned}$$

where $\Delta Y^{D}_{i}$ is the change in disposable income, and $\Delta Y^{M}_{i}$ is the change in market income for an individual *i*. Therefore, an $ASC=0.8$ would imply that 80% of a shock to the market income is absorbed by the tax-benefit system. Following this approach, we can further decompose the effect of several tax-benefit instruments, such as taxes, social security contributions and benefits. Particularly, we can analyse the impact of STW and discretionary policy measures on the income stabilisation mechanisms. We rename such a coefficient the Income Stabilisation Coefficient (ISC), as it now includes the stabilising effect of both automatic stabilisers and discretionary policy measures:

4 $$\begin{aligned}&ISC = \frac{\sum _i \Delta Y^{M}_{i} - \sum _i \Delta Y^{D}_{i}}{\sum _i \Delta Y^{M}_{i}} \nonumber \\&= \frac{\sum _i \Delta T_{i} + \sum _i \Delta SIC_{i} - \sum _i \Delta B_{i} - \sum _i \Delta STW_{i} - \sum _i \Delta DPM_{i}}{\sum _i \Delta Y^{M}_{i}}, \end{aligned}$$

where $T_i$ are taxes paid by individual *i*, $SIC_i$ social insurance contributions, $B_i$ benefits, $STW_i$ short time work and $DPM_i$ additional discretionary policy measures.

Similar approaches have been used in the literature. Paulus and Tasseva (2020) disentangle discretionary policy changes, automatic stabilisers as well as changes to market incomes and population characteristics by building on counterfactual simulations. Cantó et al. (2021) use net replacement rates and net compensation rates, to estimate the income stabilisation during the COVID-19 crisis. It should be noted that such a quantification of the income stabilisation effect rests on the implicit assumption that crisis-induced gross income changes are exogenous to the various components of the tax-benefit system. However, stabilisers might also influence gross incomes, e.g. through stabilising aggregate demand (see, among others: Bouabdallah et al., 2020). Effectively, our analysis provides a static assessment of the income stabilisation effect, while ignoring the general equilibrium dimension.

### Data

#### Calibrating the “COVID-19 scenario”

We construct our COVID-19 scenario in two main steps. Firstly, we measure the number of workers who transit to STW and unemployment in each sector of the economy during 2020. This allows us to calibrate the number of individuals who experience a labour market transition in our microsimulation model. Secondly, we estimate a probit model, which orders each individual in our micro-data by their probability of changing their labour market status. Individuals with higher probabilities will then be the ones who are transited to STW until the shares of STW in each sector of the economy are met. Such a second step allows us to account for the fact that the impact of lockdown measures was unequal across the economy for a number of reasons, including differences in home-office possibilities.

**Fig. 1:**
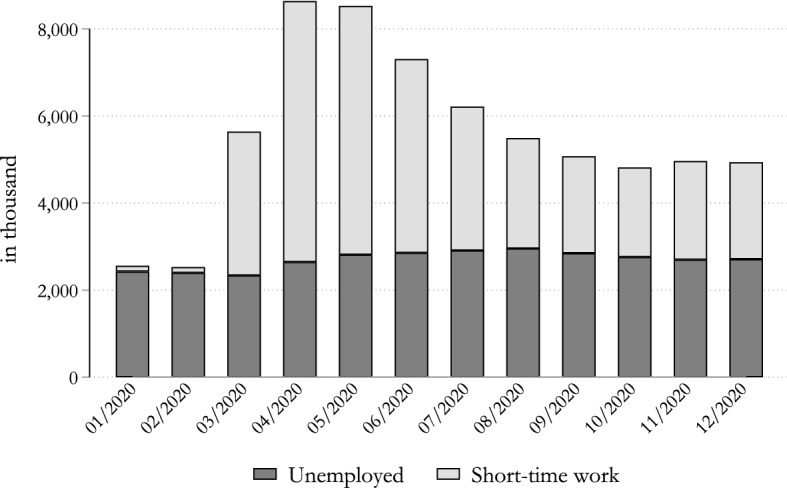
Number of workers in unemployment and STW in 2020. Note: Number (in thousands) of employees in short-time work and unemployment. Information from January to October comes from administrative data, while statistics for November and December are based on estimates from the ifo Institute. Source: ifo Institute and IAB

In step one, we calibrate the number of individuals who experience a labour market transition using administrative data from the Federal Employment Agency (“Bundesagentur für Arbeit”). These data provide us with statistics on the number of workers in unemployment and on STW.^14^ In Fig. 1, we plot the number of workers in unemployment and STW in each month of 2020. As can be appreciated from the figure, when the COVID-19 crisis hit the German labour market in March 2020, firms reacted by moving people to STW schemes. This is an expected development, given the long tradition of STW in Germany. While in February 2020 only about 133,000 workers were registered in STW, they rose sharply in March and April, with almost 6 million workers being moved to STW. Their number started decreasing in May and stabilised by the end of the year. In December, about 2.2 million employees were registered in STW. In comparison to STW, the increase in unemployment during 2020 appears less dramatic; nonetheless, its increase was significant. In March, about 2.3 million people were registered as unemployed, with this number rising to almost 3 million in August, an increase of nearly one-third.

Because of the different impact of the COVID-19 crisis and lockdown measures across the economy, STW and unemployment were not equally distributed across sectors. Table 6 in the Appendix shows data on employees in STW by sector. During the first wave of the COVID-19 crisis, the manufacturing sector was especially hit in Germany. In April, about 36% of all workers in STW schemes belonged to the manufacturing sector, followed by wholesale and retail (about 15%), and accommodation and food services (about 11%). Instead, in the third and fourth wave, it was mainly the accommodation and food service sector that was hit. In December, more than one-quarter of the workers covered by STW schemes were employed in this sector. This mainly reflects the strong lockdown measures taken by the German government in November and December, when bars and restaurants were shut down.

There is no detailed information in the administrative data on the time spent on the STW scheme by individual workers. We therefore set up a model based on survival probabilities to obtain estimates for the duration of individuals in this schemes (in months). For this purpose, we use the information on the number of individuals in STW in each month and assume that the total number of people entering in 2020 is indicated by the month with the highest share of individuals in STW (April). For any consecutive months, we then postulate that some people left STW schemes and no new persons entered into the scheme. Since in the second wave of COVID-19, in November and December 2020, we observe a small increase in the number of persons in STW, we assume that employees that have already been in STW in the previous months are re-entering the scheme.

On top of the duration of STW schemes across sectors, we need information on the reduction of hours for workers in STW. Therefore, we use information provided by the Federal Employment Agency on the duration of people in STW over 2020 and report them in Table 1. This information is available also by sector. There we can appreciate that about 31.8% of workers in STW schemes reduced their hours only slightly (by less than 25%), 38.9% worked only about 25 to 49%, 18.1% reduced working time by between 50 and 74% and 9.8% by between 75 and 99%. Additionally, only a few workers (1.3%) reduced their working hours to zero.

**Table 1:** Distribution of hours reduction for workers in STW in 2020

Hour reduction	<25%	25–49%	50–74%	75–99%	100%
Total	31.8%	38.9%	18.1%	9.8%	1.3%
Manufacturing	48.6%	41.0%	8.0%	2.2%	0.2%
Construction	41.9%	33.8%	15.3%	7.8%	1.1%
Wholesale and retail	31.0%	41.9%	17.4%	8.6%	1.2%
Transport and storage	21.8%	35.7%	31.5%	10.0%	1.0%
Accommodation and food services	9.1%	31.0%	31.2%	24.9%	3.9%
Information and communication	33.0%	41.2%	16.8%	7.8%	1.1%
Professional, scientific, ... Activities	26.4%	44.3%	18.9%	9.0%	1.3%
Administrative and Support Services	11.5%	36.2%	32.2%	18.4%	1.7%
Rest	21.9%	38.2%	22.1%	15.6%	2.1%

Note: Share of people in short-time work with a reduction in the number of hours worked by less than 25%, between 25% and 49%, between 50% and 74%, between 75% and 99%, and 100% (working 0 hours). Source: Own calculation based on data from the Federal Employment Agency (“Bundesagentur für Arbeit”)

After calibrating the number of workers that experience a labour market transition to STW and unemployment, and the reduction of working hours for those in STW, we select the individuals who make a transition. We therefore estimate a probit model that allows us to identify in our micro-data those individuals that are more likely to change their labour market status in response to the COVID-19 crisis. For this purpose, we use administrative data obtained by the Institute of Employment Research (IAB) through the online survey “Leben und Erwerbstätigkeit in Zeiten von Corona (HOPP)”.^15^ This is a representative random sample, including individuals at least 18-years-old at the time of the interview. The data were collected monthly between May and September 2020. We use the total information of these four waves of the survey to estimate the average probability across the year 2020 to transit to STW. The sample is drawn from register data, which allows us to add additional information related to the characteristics of individuals. For a more detailed description and a summary of the HOPP survey, see Haas et al. (2021) and Sakshaug et al. (2020). The IAB then used the HOPP data to estimate a probit model that estimates the probability of being in STW schemes, given the characteristics of individual workers.^16^ Table 2 shows the average marginal effects of the probit estimation for Germany and highlights that there are substantial differences across individuals.^17^

Similar to what Brewer and Tasseva (2021) found for the UK, also in Germany, individuals in low-income households are significantly more likely to transit to STW. A disposable household income below 1500 euro monthly increases the probability of being in STW by almost 10 percentage points compared to the reference category (someone with a disposable household income between 2,000 and 3,000 euro). Additionally, women are less likely to be in STW compared to men. Also, there seems to be no significant difference in the probability of being in STW across age groups, except for people above 60, who are less likely to be in STW. Having a partner, as well as having a foreign citizenship, also significantly increases the probability for people to be in STW.

**Table 2:** Probability of being in STW (Probit model - average marginal effects)

Variables	Marginal effect	SE
Household disposable income (ref: 2000–3000 Euro)		
Below 1500 euro	0.104***	0.028
1500–2000 euro	0.027	0.020
3000–4000 euro	−0.035***	0.014
4000–5000 euro	−0.066***	0.014
5000 euro or more	−0.076***	0.014
Gender (ref: male)	−0.039***	0.008
Age (ref: 40–49)		
18–29	−0.003	0.017
30–39	0.016	0.013
50–59	0.010	0.012
60 or above	−0.026*	0.015
Partner (ref: no)	0.033***	0.011
Children (ref:no)	0.010	0.010
Education (ref: upper-secondary)		
Primary or below	0.207	0.126
Lower-secondary	0.009	0.014
Post-secondary	0.024	0.016
Tertiary	−0.036***	0.013
Citizenship (ref: only German)		
German and other	0.018	0.030
Other	0.066**	0.028
Observations	16,053	

Note: */**/*** means significant at 10%/5%/1% level; Reading example: A marginal effect of −0.039 for females means that cet. par. women are 3.9 percentage points less likely to be in STW than the reference category (men). Source: Calculations by the Institute for Employment Research (IAB), based on HOPP Panel (Hochfrequentes Online-Personen-Panel “Leben und Erwerbstätigkeit in Zeiten von Corona”, see Haas et al. (2021))

Based on the estimated coefficients of the probit model, we predict the probability of each individual in the EU-SILC data being in STW (adding a random error term). Instead of randomly choosing people that transit to STW schemes in each sector, we chose according to our probit model. In more detail, we order individuals based on the difference between their predicted probability of moving to STW and a random draw from a uniform distribution (this difference can be positive or negative). Starting from individuals whose difference is the largest, we then select individuals to move to STW until the target share by sector (i.e. the share observed in the administrative data) is reached. As a result, we proportionally move more low-income earners as well as more males and individuals without children to STW. It is worth mentioning that this modelling choice has substantial consequences for the results presented later. In Appendix 1, we highlight the differences compared to choosing individuals at random by sector (as done in previous work) in more detail.

On the other hand, the low number of individuals observed as new unemployed in the HOPP data do not allow us to estimate a probit model on the probability to enter in unemployment. Therefore, workers moving to unemployment are selected based on a random assignment by sector of activity.^18^

To macro-validate our model calibration, we estimate the increase in the costs for STW schemes when modelling the transition on the labour market. Our model suggests an additional cost for the government of about 19.7 billion euro. The Federal Employment Agency (“Bundesagentur für Arbeit”) reported costs of about 21 billion euro, which is slightly above our estimates.^19^

Additionally, we validate our model by comparing the share of workers that we transit to STW by income group with the one observed in the HOPP data.^20^ As Table 3 highlights, our model performs well in this dimension, although we slightly overestimate the share of low-income households and slightly underestimate the one of high income households; differences are nonetheless minor.

**Table 3:** Model validation

	Probit model (EU-SILC) (%)	HOPP data (%)
Below 1500	11.3	7.4
1500–2000	9.1	9.3
2000–3000	24.7	24.9
3000–4000	22.1	22.7
4000–5000	15.7	16.5
5000+	17.2	19.2
Total	100.0	100.0

#### Calibrating the “COVID-19 scenario w/o STW and DPM”

For this scenario without the STW scheme and DPM, we model the same shock on the labour market as in the COVID-19 scenario. Namely, we impose the same reduction of working hours, but we assume that workers who would have been transited to STW move to unemployment instead. Given that for unemployed individuals the hours reduction is complete, we translate the reduction of working hours into full-time equivalents (FTE) jobs that are lost by sector. Therefore, differently from the scenarios where STW are in place, the same reduction of working hours is no longer spread among a broader set of workers but rather concentrated among fewer individuals who become fully unemployed.

It should be noted that, in doing so, we are effectively assuming that the working hours reduction due to the COVID crisis would have been the same without the income support provided by STW and DPMs. Given the role of STW in avoiding a spiral of declining employment, wages and hence aggregate demand and output (see Boeri & Bruecker, 2011), this assumption is likely to result in a conservative estimation of the income cushioning effect of these measures.^21^

## Results

We now move to present the main results of our analysis. In Sect. 4.1, we analyse the impact of the COVID-19 crisis on household income and investigate the cushioning effects of the STW scheme and DPM. In Sect. 4.2, we then turn to explore the stabilising role played by the German tax-benefit system during the COVID-19 crisis. We decompose this stabilisation effect and analyse in greater detail the role played by specific policy instruments. Finally, in Sect. 4.3, we analyse the impact of the COVID-19 crisis on inequality and poverty in Germany.

### The cushioning effect of STW and DPM

In this subsection, we investigate the impact of the COVID-19 crisis on German household income and explore the income cushioning effect of STW and of DPM. To measure the impact of COVID-19 on German household income, we compare different COVID-19 scenarios with the no-COVID-19 scenario, which assumes no DPM nor labour market changes due to the COVID-19 crisis (see description in Sect. 3.1.2).

**Fig. 2:**
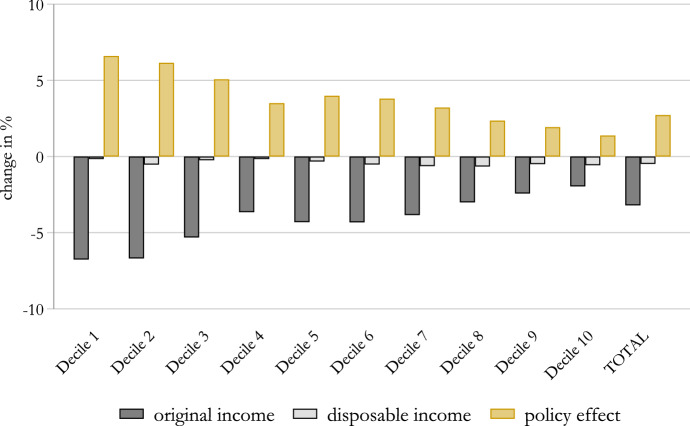
Impact of the COVID-19 crisis on household income. Note: Percentage change in household market and disposable income by income deciles. Income deciles are based on the baseline (no-COVID-19 scenario) distribution of equivalised disposable income. The equivalent income is calculated based on the modified OECD scale. Source: Own calculations using EUROMOD I3.0+

In Fig. 2, we plot the percentage changes in market income and disposable income under the COVID-19 scenario with respect to the no-COVID-19 scenario. We observe that the COVID-19 crisis caused a significant reduction of market income across the whole spectrum of the income distribution, with an overall drop of almost 3$\%$. This effect is largely regressive, with households in the lowest deciles of the income distribution losing a substantially higher share of their market income than those in the highest deciles. This is not surprising given that higher-skilled workers have been far less disrupted by the COVID-19 crisis (e.g. because they can work from home, their sectors of activities have been less exposed to the crisis, etc.).

Once taxes and benefits are accounted for, the drop in income is greatly mitigated, with an average fall in disposable income in the order of $0.5\%$. However, the regressive effect is only partially reversed. Indeed, the impact on disposable income is somehow flattened across the distribution. Our estimated impact is close to the one estimated by Bruckmeier et al. (2021), who predict a fall in disposable income of a similar magnitude. However, differently from us, they find the effect of the COVID-19 crisis to be slightly progressive. In this regard, it should be noted that this would have also been the case in our analysis had we selected at random the individuals who experience the labour market transition in each sector of the economy (see Fig. 5 in the Appendix). Instead, by using a probit model, we can account for the fact that individuals in the lower part of the income distribution are more likely to enter in the STW schemes.^22^ This shows the importance of accounting for a broader set of worker characteristics when simulating labour market transitions in micro data instead of using the sector of activity alone.^23^

We now turn to explore the contribution of STW and DPM in cushioning the effect of the COVID-19 crisis on household income. For this purpose, we construct a counterfactual scenario without the STW scheme and the DPM. We then compare it with the COVID-19 scenario where these policies are instead in place (see the detailed description of the scenarios in Sect. 3.1.2).

**Fig. 3:**
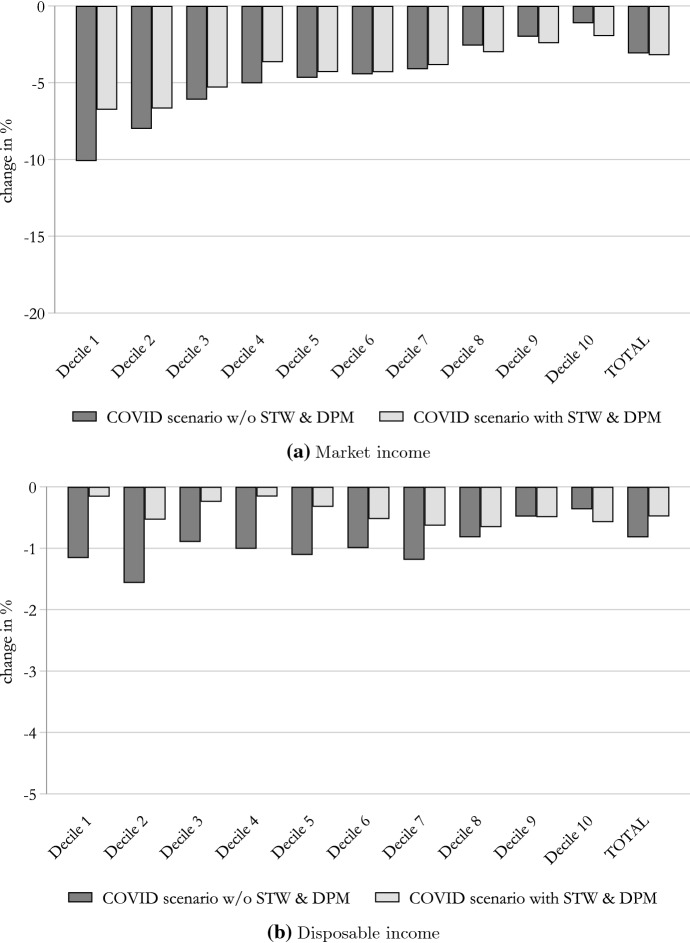
Impact of the COVID-19 crisis on household income. Note: Percentage change in household market and disposable income by income deciles. Income deciles are based on the baseline (no-COVID-19 scenario) distribution of equivalised disposable income. The equivalent income is calculated based on the modified OECD scale. Source: Own calculations using EUROMOD I3.0+

In Fig. 3, we analyse the impact of the COVID-19 crisis on household market income (panel a) and disposable income (panel b) for the scenario with STW and DPM as opposed to the scenario where these are not in place.

Starting with market income (Fig. 3a), we observe that its total reduction is similar in both scenarios; that result is expected given that we are assuming the same reduction of hours of work. However, when STW and DPM are not in place, the income loss is substantially stronger in the lowest deciles of the distribution. That is because, without STW, hours of work reductions are no longer spread among a broader set of workers but rather concentrated among fewer individuals who become fully unemployed (i.e. workers who are laid off cannot have their working hours only partially reduced). Those individuals are mostly concentrated in the lowest deciles of the distribution because our probit model typically assigns low-skill/low-income workers a higher probability of changing labour market status.

Moving to analyse the effect on disposable income (Fig. 3b), we can appreciate that in both scenarios the tax-benefit system largely offsets the impact of the COVID-19 crisis on households. However, in the COVID-19 scenario (with STW and DPM), the regressive impact of the crisis is largely reversed, while this is not the case in the scenario where these are not in place. In particular, without STW and DPM, the impact on disposable income remains largely regressive, with the lowest three deciles suffering an income reduction that is twice as large. This highlights the central role of STW and DPM in cushioning the income of poorer households.

### Income stabilisation in times of COVID-19

Having analysed the role of STW and DPM in cushioning the effects of the COVID-19 crisis, we now turn to explore the contribution of the various components of the tax-benefit system in stabilising household income. For this purpose, we calculate the income stabilisation coefficient (ISC), as set out in Sect. 3.1.3, for our COVID-19 scenarios with and without STW and DPM. This indicator is calculated on the entire population and it allows us to assess the effectiveness of the German tax-benefit system and of the DPM as automatic stabiliser.

In more detail, we decompose the income stabilisation coefficient into five main components, including: (i) taxes (including social security contributions), (ii) unemployment benefits, (iii) STW, (iv) discretionary policy measures and (v) other benefits (including pensions).

**Fig. 4:**
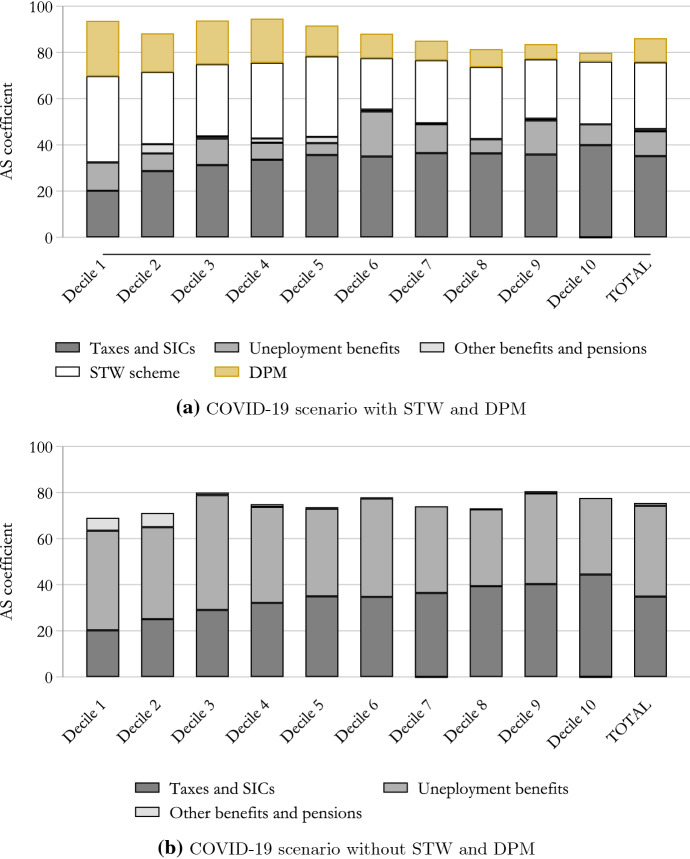
Income stabilisers during the COVID-19 crisis. Note: Income deciles are based on the baseline (no-COVID-19 scenario) distribution of equivalised disposable income. The equivalised income is calculated based on the modified OECD scale. Source: Own calculations using EUROMOD I3.0+

**Fig. 5:**
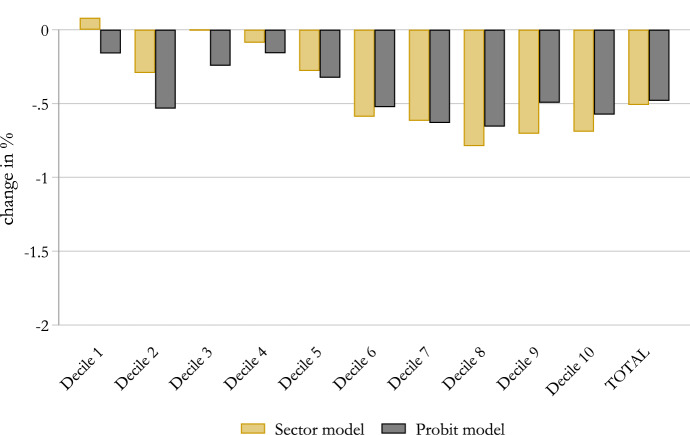
Impact of the model choice on disposable income. Note: Income deciles are based on the baseline (no-COVID-19 scenario) distribution of equivalised disposable income. The equivalent income is calculated based on the modified OECD scale. Source: Own calculations using EUROMOD I3.0+

Figure 4 shows the stabilisation coefficient and its breakdown for the COVID-19 scenario with STW and DPM and for the scenario where they are not in place (respectively, in panel a and panel b). Starting with 4a, we can appreciate that the German tax-benefit system, including STW and DPM, was able to absorb about 85% of the income shock caused by the COVID-19 crisis in 2020 in total. In other words, a loss of 100 euro in market income only translated into a loss of 15 euro of disposable income. Moreover income stabilisation, ranging between 93% in the lowest decile to 79% in the highest decile, was stronger for low income earners indicating that the tax-benefit system in Germany protected poorer households more than richer ones. Looking at the income stabilisation by individual components of the tax-benefit system, it appears that for poorer households this is largely driven by STW and DPM, whereas for richer households the reverse effect of the progressive income taxation plays the most important role. The DPM, in particular the one-off payment for households with children, absorb a higher share of income loss in the first decile, due to the nature of this benefit (lump-sum) and the lower market income loss in relative terms, experienced by households in the first decile.

Moving to 4b, we analyse what the income stabilisation capacity of the German tax-benefit system would have been had STW and DPM not been in place. We find that the income stabilisation capacity substantially reduces, especially for low-income earners. In more detail, the ISC drops to about 69% for low-income earners. Also, for households in the middle of the income distribution, the stabilisation effect drops below 80% (ranging between 73% and 77%). This is explained by the fact that individuals in the lower decile are more likely to have a discontinuous working history. Therefore, in the COVID-19 scenario without STW and DPM they might be not eligible for unemployment benefit. Moreover, the lack of DPM (mainly the COVID-related child benefit) and the slightly lower income stabilisation that unemployment benefits offers compared to STW schemes, appears to play a significant role.

Overall, our analysis suggests that income stabilisers were effective in cushioning the income loss caused by the COVID-19 crisis in Germany in 2020, and that, as far as low-income earners are concerned, STW and DPM played a key role.

### The impact of the COVID-19 crisis on inequality and poverty

In this subsection, we consider the impact of the COVID-19 crisis on inequality and poverty. We begin analysing the impact of COVID-19 on inequality measures in Table 4. There we can observe the Gini coefficient for four different income concepts, from market income (A) to disposable income inequality (D). Consistent with our findings on the impact of household income, we find that the Gini of market income features a significant increase of about 0.9 percentage points. COVID-19 has therefore led to a large increase in income inequality before taxes and benefits are accounted for. In the absence of policy measures, the increase would have been even stronger, and of about 1.4 percentage points.

**Table 4:** Impact of the COVID-19 crisis on inequality

	Inequality across scenarios			Diff. w.r.t. Baseline			
	Baseline	COVID-19 (w/o)	COVID-19 (with)	COVID-19 (w/o)		COVID-19 (with)	
Gini							
A = market income	0.5056	0.5200	0.5144	0.0144	(0.0010)	0.0088	(0.0008)
B = A - taxes and SIC	0.5379	0.5554	0.5487	0.0175	(0.0011)	0.0108	(0.0009)
C = B + pensions	0.3171	0.3316	0.3248	0.0144	(0.0010)	0.0077	(0.0008)
D = C + benefits (disp. inc)	0.2762	0.2797	0.2763	0.0036	(0.0005)	0.0002	(0.0004)
Additional measures							
Redistribution index	0.2295	0.2402	0.2380	0.0108	–	0.0086	–
Quantile share ratio (S80/S20)	4.0688	4.1145	4.0606	0.0456	–	−0.0083	–
Inter-decile ratio (D5/D1)	1.8602	1.8996	1.8683	0.0394	–	0.0081	–

Note: We show results for 3 different scenarios: “baseline”: no-COVID-19 scenario; “COVID-19 (w/o)”: COVID-19 scenario without STW and DPM; “COVID-19 (with)”: COVID-19 scenario (with STW and DPM). Gini coefficients are based on equivalised income using the modified OECD scale. Standard Errors (SE) reported in brackets.Source: Own calculations using EUROMOD I3.0+

As taxes and benefits are introduced into the equation (see Gini B to D), we note that taxes do not seem to play an important role in closing this gap; the benefit system on the other hand has the effect of largely cushioning the increase in market income inequality caused by the COVID-19 crisis. Therefore, while inequality in market income increases significantly, the effect on disposable income inequality is almost zero, highlighting the importance of the benefit system in protecting poorer households.

On the other hand, if we compare the impact of the COVID-19 crisis in the absence of STW schemes and DPM, we see that the Gini coefficient of disposable income would have risen substantially (0.4 percentage points). This shows the importance of STW schemes and DPM in protecting against a substantial increase of inequality. Our results highlight that in Germany the tax-benefit system is able to offset the increase in income inequality completely.

Table 4 shows additional inequality measures, beyond the Gini coefficient. The quantile share ratio as well as the inter-decile ratio confirm the insights offered by the Gini, particularly the inequality cushioning effect of STW schemes and DPM.

Finally, we consider the impact of the COVID-19 crisis on poverty risks. Table 5 presents the at-risk-of-poverty (AROP) rate (using $60\%$ of median equivalised household disposable income as the poverty line) for various household types, both for the scenario with and without the COVID-19 crisis.

**Table 5:** Impact of the COVID-19 crisis on poverty

Household type	Poverty across scenarios			Diff. w.r.t. Baseline			
	Baseline	COVID-19 (w/o)	COVID-19 (with)	COVID-19 (w/o)		COVID-19 (with)	
One adult <65, no children	28.4	29.7	29.4	1.2	(0.2)	1.0	(0.2)
One adult $\ge$65, no children	26.2	26.4	26.4	0.2	(0.1)	0.2	(0.1)
One adult with children	38.5	39.2	36.9	0.7	(0.4)	−1.6	(0.8)
Two adults <65, no children	11.2	11.8	11.3	0.6	(0.2)	0.2	(0.2)
Two adults, at least one $\ge$65, no children	10.1	10.3	10.3	0.2	(0.1)	0.2	(0.1)
Two adults with one child	7.9	9.0	8.4	1.1	(0.5)	0.5	(0.3)
Two adults with two children	4.9	6.8	5.2	1.9	(0.6)	0.3	(0.4)
Two adults with $\ge$3 children	11.5	13.5	10.9	1.9	(1.5)	−0.6	(1.8)
$\ge$Three adults, no children	6.3	6.5	6.4	0.2	(0.2)	0.1	(0.1)
$\ge$Three adults with children	6.2	7.5	6.8	1.3	(0.8)	0.6	(1.0)
All	14.0	14.9	14.2	0.9	(0.1)	0.2	(0.1)

Note: We show results for 3 different scenarios: “baseline”: no-COVID-19 scenario; “COVID-19 (w/o)”: COVID-19 scenario without STW and DPM; “COVID-19 (with)”: COVID-19 scenario (with STW and DPM). Poverty line is EUR 14,430.48 (60% of median equivalised annual disposable income) anchored to the value of the baseline. Standard Errors (SE) reported in brackets. Source: Own calculations using EUROMOD I3.0+

The overall AROP rate in Germany is about 14.0% in our baseline scenarion (no-COVID-19), with one parent households (38.5%) and single households (between 26.2% and 28.4%) displaying the highest AROP rates. On the contrary, households with more than one adult (including those with more children) have lower poverty rates. The labour market shock caused by COVID-19 is expected to increase these poverty rates substantially. In the COVID-19 scenario without STW and DPM, the overall AROP rate increases significantly to 14.9%. The increase is particularly large for families with children, the increase in poverty is significant (+1.9 pp). But also for single-person households of working age we observe a significant increase (i.e. 1.2 pp), mostly because when these individuals lose their jobs, they cannot count on income received by other household members. Even in the COVID-19 scenario where STW and DPM are in place, the overall AROP rate increases, although to a substantially smaller extent (14.2%). In this scenario, AROP rates for families with more than 2 children and for single-parent families do not increase. This result is expected given they are the target groups of the discretionary policy measures (child bonus and a higher tax allowance for single-parents) and because of the higher replacement rate of STW for individuals with children. It suggests that discretionary policy measures were essential in protecting single-parent households, which is the group with the highest AROP.

Altogether, our findings suggest that, in spite of the regressive nature of the COVID-19 crisis, STW and DPM have largely offset its impact of the pandemic on inequality and poverty.

## Conclusion

In this paper, we employ EUROMOD to analyse the impact of the COVID-19 crisis on German households. In particular, we use detailed up-to-date administrative data on STW and unemployment, together with labour market transition techniques, to model the impact of the COVID-19 crisis on household income and, therefore, on inequality and poverty. Additionally, by setting up a hypothetical scenario where STW and DPM are not in place, we are able to evaluate the cushioning effect of these policies during the COVID-19 crisis in Germany.

Our analysis estimates that German households lost more than 3% of their market income in 2020, due to the COVID-19 crisis. The effect was regressive and households in the lower part of the income distribution were affected more severely because low-income earners are more likely to enter in STW schemes. However, the fall in market income was largely offset by the tax-benefit system, which softened the reduction in disposable income to a more modest 0.5%. Indeed, the German tax-benefit system, together with the DPM introduced in response to the COVID-19 crisis, are estimated to absorb about 85% of the income shock, with a stronger stabilisation for low income earners.

Our study highlights the importance of STW and DPM (the COVID-19 one-off child benefit and the increase in the tax allowance for single parents) in cushioning the impact of the COVID-19 crisis. These policies play a crucial role in income stabilisation for low-income earners, therefore counteracting the expected increase in inequality and at-risk-of poverty in 2020. This is especially true for single-parent families and for households with more than two children who benefit from the DPM, as well as from the higher replacement rate of STW for individuals with children. The strong income stabilising property of STW and DPM for low incomes might also help to overcome a strong reduction in household demand, since liquidity constrained households are typically more present in the lower part of the income distribution.

This work contributes to the literature of modelling the socio-economic impacts of the COVID-19 pandemic by showing that the modelling is key for the estimation results. The absence of real-time data can lead to severe problems in evaluating the impact of such a crisis, especially when it comes to income inequality. Contrary to the results found using traditional approaches (which are based on either re-weighting or stochastic labour market transitions), we find that the impact of the COVID-19 crisis on disposable household income is quite similar in size across the income distribution (between 0.2 and 0.6%). Therefore, using the extended labour market transition approach provides an important technique also for the analysis of future macroeconomic shocks, because it allows to account for worker characteristics when simulating labour market transitions in micro-data.

Comparing our results with similar work in other countries, we find that discretionary policy measures in Germany are slightly less effective in cushioning household income, compared to Austria, where Christl et al. (2021b) estimated the ICS of 87% with a similar approach. The results differ especially along the income distribution, where in Austria, the cushioning effect of the tax-benefit system was substantially higher for low-income earners, and lower for high-income earners. Additionally, Cantó et al. (2021) calculated different income stabilising indicators for Spain, Belgium, the UK and Italy. In comparison to Germany, only Belgium seems to provide similar protection of households against an income loss.^24^ Overall, the results from Germany highlight the importance of having strong automatic stabilisers (e.g. through STW) in place to cushion income losses during macroeconomic crises.

## Appendix

### Appendix 1: Selection of short-time workers

There are two approaches taken in the literature to define people that are moving to STW. In the first approach, detailed aggregate statistics on the amount of workers in STW schemes are used (usually by sector) based on the argument that a sector-specific random allocation (or even more detailed components) might cover well the heterogeneity in characteristics of workers that transit to STW schemes.^25^ Since the COVID-19 shock in 2020 was strongly defined by a sectorial component that was mainly driven by lockdowns that hit specific sectors more than others, such as tourism, hotels and bars, this assumption seems to be reasonable on first glance. On the other hand, other approaches, such as that employed by Brewer and Tasseva (2021), use specific micro-data to estimate the probability of a worker moving to STW schemes. This approach allows for the estimation of the risk of a worker moving to short-time work.

We compare both approaches in our paper on Germany using the probit model (discussed in Sect. 3.2) to analyse the impact of the modelling choice related to transition to STW. We compare the random allocation by sectors with the allocation where those workers with the highest risk of being sent to STW transit. Figure 5 already highlights the strong distributional impact of the modelling assumption. While according to the probit model low-income earners have a higher risk of being sent to STW schemes, the sectorial approach, where people are randomly sent to STW in each sector, does not cover this fact well.

As we have shown, when using the detailed information on worker heterogeneity in STW schemes, the effect on disposable income is regressive. By contrast, when using the simpler sector approach, as often used in the literature, the effect turns out to be progressive. Our results clearly highlight the importance of the modelling choice in controlling for characteristics of those people that move to STW schemes when estimating the impact of the pandemic on household income and inequality.

### Appendix 2: Additional tables and graphs

See Tables 6 and 7.

**Table 6:** Number and share of people in STW across sectors in Germany 2020

	March	April	May	June	July	August	September	October	November	December
Manufacturing	1,100,157	1,869,069	2,027,961	1,773,324	1,390,025	1,038,743	927,287	835,298	723,663	611,261
	(33.3%)	(31.2%)	(35.5%)	(39.8%)	(42.0%)	(41.0%)	(41.6%)	(40.6%)	(32.0%)	(27.4%)
Construction	77,071	150,741	122,262	88,711	64,878	52,083	46,362	44,950	46,188	45,563
	(2.3%)	(2.5%)	(2.1%)	(2.0%)	(2.0%)	(2.1%)	(2.1%)	(2.2%)	(2.0%)	(2.0%)
Wholesale and retail	530,070	1,035,969	841,670	594,690	403,587	299,785	265,669	232,683	241,709	260,446
	(16.0%)	(17.3%)	(14.7%)	(13.4%)	(12.2%)	(11.8%)	(11.9%)	(11.3%)	(10.7%)	(11.7%)
Transport and storage	183,856	331,524	319,738	256,269	200,354	173,248	152,387	148,884	142,613	147,834
	(5.6%)	(5.5%)	(5.6%)	(5.8%)	(6.1%)	(6.8%)	(6.8%)	(7.2%)	(6.3%)	(6.6%)
Accommodation and food services	367,182	665,678	634,970	445,667	321,536	252,673	219,530	233,151	526,767	574,332
	(11.1%)	(11.1%)	(11.1%)	(10.0%)	(9.7%)	(10.0%)	(9.9%)	(11.3%)	(23.3%)	(25.8%)
Information and communication	79,682	130,998	151,255	135,588	111,346	89,909	75,349	62,923	46,862	63,041
	(2.4%)	(2.2%)	(2.6%)	(3.0%)	(3.4%)	(3.5%)	(3.4%)	(3.1%)	(2.1%)	(2.8%)
Professional& scientific activities	243,981	454,831	409,409	321,646	238,737	192,734	164,963	148,029	135,331	120,445
	(7.4%)	(7.6%)	(7.2%)	(7.2%)	(7.2%)	(7.6%)	(7.4%)	(7.2%)	(6.0%)	(5.4%)
Administrative services	233,033	424,609	400,852	321,515	248,913	202,353	183,335	175,078	161,866	167,685
	(7.0%)	(7.1%)	(7.0%)	(7.2%)	(7.5%)	(8.0%)	(8.2%)	(8.5%)	(7.1%)	(7.5%)
Rest	490,856	932,010	806,725	514,875	326,511	233,895	191,800	177,602	239,272	238,637
	(14.8%)	(15.5%)	(14.1%)	(11.6%)	(9.9%)	(9.2%)	(8.6%)	(8.6%)	(10.6%)	(10.7%)
Total	3,305,887	5,995,428	5,714,842	4,452,285	3,305,887	2,535,423	2,226,680	2,058,598	2,264,269	2,229,244

Source: Federal Employment Agency (“Bundesagentur für Arbeit”) and ifo Institute

**Table 7:** Average equivalised market and disposable income by income deciles

Decile	Market income			Disposable income		
	Baseline	COVID-19 (with)	COVID-19 (w/o)	Baseline	COVID-19 (with)	COVID-19 (w/o)
1	3739	3484	3363	9789	9791	9673
2	10,452	9749	9596	14,670	14,587	14,415
3	13,715	13,003	12,896	17,607	17,589	17,451
4	17,091	16,472	16,212	20,224	20,213	20,005
5	21,326	20,392	20,328	22,688	22,627	22,423
6	26,768	25,565	25,544	25,516	25,395	25,261
7	32,154	30,955	30,854	28,603	28,452	28,272
8	38,635	37,494	37,668	32,491	32,284	32,222
9	48,180	47,014	47,235	38,527	38,344	38,348
10	89,574	87,833	88,547	61,016	60,668	60,786

Source: Own calculations using EUROMOD I3.0+

## Footnotes

1 In comparison to STW, the increase in unemployment during 2020 was less dramatic. In August 2020, the number of unemployed rose to almost 3 million, compared to about 2.3 million in March of the same year.

2 Country-specific analyses have been conducted by Christl et al. (2021b) for Austria, Marchal et al. (2021) for Belgium, Doorley et al. (2021) for Ireland and Brewer and Tasseva (2021) for the UK.

3 Special thanks to Christopher Osiander and Thomas Kruppe for providing us with the relevant information.

4 To be precise, the approach of Bruckmeier et al. (2021) combines five different tools: (i) a VAR model of output shocks at the industry-level, (ii) a structural labour demand model, (iii) estimated information on employment changes by industry and worker type (by interacting the estimated industry-level output shocks with output elasticities from the labour demand model), (iv) a maximum entropy-based approach to feed these predicted shocks to household micro-data, and (v) a microsimulation model to assess the distributional consequences. Elements (i) and (iv) are new compared to the approach of Bargain et al. (2012). In our approach, we do not need steps (i) and (ii) as we use administrative data on the use of the STW scheme which directly gives us the shocks from step (iii). We also use a different method (step iv) to translate these shocks to the microdata by using a probit model (based on a broad set of worker characteristic) instead of a random selection procedure.

5 This is in line with the results of Alipour et al. (2020), who found that low-skilled and low-wage earners are most vulnerable to the consequence of the COVID-19 crisis.

6 More detail can be found in the EUROMOD Country Report for Germany.

7 Other discretionary policies included the emergency child benefit supplement for parents who lost earnings because of the COVID-19 pandemic, as well as the simplified access to basic income support and support to cover the cost of heating and housing for low-income families. We could not simulate these benefits in EUROMOD due to limited information in the data. However, these measures are small compared to the other discretionary policy measures simulated.

8 For more details, see the EUROMOD Country Report for Germany.

9 Again, for more details on these policies and on their simulation in EUROMOD, we refer the interested reader to the EUROMOD Country Report for Germany.

10 Self-employed or small firms with up to five employees can apply for a maximum of 9,000 euro, and firms with up to 10 employees can apply for a maximum of 15,000 euro.

11 For the details about the uprating factors used in each case, refer to the latest EUROMOD country report for Germany.

12 See also the Summary Note For EUROMOD: Labour Market Adjustment Add-on.

13 Because our main interest is to analyse the net flow from employment to unemployment, as well as the cushioning effect of STW and discretionary policy measures, we do not include transitions to employment.

14 Given that information on STW for November and December are not yet available, we complement these data with estimates from the ifo Institute. See ifo Institute (2021).

15 See Volkert et al. (2021).

16 See also Kruppe and Osiander (2020) for an estimation for May 2020, detailed data description can be found in Haas et al. (2021).

17 Please note that the results for 2020 are very similar to the results obtained in each subsample in 2020. The probit estimates are very stable across the year 2020.

18 Alternatively, we could have assumed that the characteristics of the individuals moving to unemployment are similar to those of the individuals entering in STW. However, this is a strong assumption and given that the transition to unemployment affects a lower number of individuals, the modelling choice is expected to have only a minor impact on the results.

19 See Federal Employment Agency (2021).

20 Note again that we know from administrative data how many workers in each sector moved to STW. We use this information, in combination with our probit model, to move people to STW. We do not show aggregates by sector, because by definition, our model chooses exactly the correct number of people by sector.

21 On the other hand, one could argue that the presence of STW schemes might lead to an excess of hours reduction, for they involve the same moral hazard problem arising in the provision of unemployment insurance. i.e. workers and employers may collude in extracting payments from the state. However, Boeri and Bruecker (2011) note that this issue is less present during downturns and less relevant in the German system.

22 This is in line with the insights of Alipour et al. (2020) who found that low-skilled and low-wage earners are most vulnerable to the consequence of the COVID-19 crisis.

23 Note, however, there are many more differences of our approach compared to the one of Bruckmeier et al. (2021) including type and scope of data used, detailed inclusion of STW based on administrative data instead of a macro-model-based approach as well as a different microsimulation model. This makes it hard to single out the exact reasons for the differences in results.

24 Please note that the methodology, as well as the time line (only one month is analysed) is different in the income stabilising coefficients estimated by Cantó et al. (2021).
